# Immune checkpoint inhibitor-related adverse cardiac events in patients with lung cancer: a systematic review and meta-analysis

**DOI:** 10.1186/s12935-022-02760-2

**Published:** 2022-11-19

**Authors:** Xiao-Tong Zhang, Nan Ge, Zi-Jian Xiang, Tao Liu

**Affiliations:** 1 Department of Pulmonary and Critical Care Medicine, Peking Union Medical College Hospital, Chinese Academy of Medical Sciences and Peking Union Medical College, No.1 Shuaifuyuan Wangfujing Dongcheng District, Beijing, 100730 China; 2 Department of Geriatrics, Peking Union Medical College Hospital, Chinese Academy of Medical Sciences and Peking Union Medical College, Beijing, China; 3 Beijing Zhiyun Data Technology Co. LTD, Beijing, China

**Keywords:** Immune checkpoint inhibitor, Immunotherapy, Cardiotoxicity, Myocarditis, Immune related adverse event

## Abstract

**Background:**

Although people are more and more aware of the cardiotoxicity caused by immune checkpoint inhibitors (ICIs) in the treatment of lung cancer, its incidence rate has not been systematically analyzed. This study aims to evaluate the incidence of cardiotoxicity related to the ICI therapies for lung cancer, so as to enhance clinicians' attention to cardiotoxicity, implement proper prevention and intervention for high-risk patients, and minimize the risk of cardiac dysfunction during and after completion of therapy.

**Methods:**

We conducted a systematic literature search for relevant publications in PubMed and Scopus from inception to 19 April 2022. Pooled incidence and risk ratios with 95% confidence intervals (95% CIs) for cardiotoxicity events were calculated.

**Results:**

A total of 37 studies covering 38 trials, including 14,342 patients, were identified. The pooled risk ratios of incidence of any cardiac AEs were 1.944 [95% CI 0.8–4.725] (Single ICI versus chemotherapy), 1.677 [95% CI 1.065–2.64] (Single ICI plus chemotherapy versus chemotherapy), and 0.478 [95% CI 0.127–1.798] (Single ICI versus Dual ICI). The incidence of myocarditis and arrhythmia were 0.003[95%CI 0.002–0.006] and 0.014[95%CI 0–0.037], respectively.

**Conclusion:**

Single ICI did not increase the risk of cardiotoxicity compared with chemotherapy, and single ICI plus chemotherapy increased the risk of cardiotoxicity by 67% compared with chemotherapy alone. Combination immunotherapy did not increase the risk of cardiotoxicity compared with single ICI.

**Supplementary Information:**

The online version contains supplementary material available at 10.1186/s12935-022-02760-2.

## Introduction

Patients with lung cancer, especially with advanced or metastatic lung cancer, are often poorly treated due to high morbidity and mortality [1]. The treatment prospects of this refractory disease, however, have changed with the in-depth research on immune checkpoint inhibitors (ICIs) in recent years [2]. Immune checkpoints are immunosuppressive molecules that protect human tissues and organs by regulating the immune response to maintain tolerance. They are monoclonal antibodies that prevent these molecules from releasing the immune system and killing tumor cells [3], including PD-1, PD-L1 and CTLA-4. As ICIs are widely used in the treatment of lung cancer, especially metastatic and advanced lung cancer [4], an excessively enhanced immune response has led to a wide range of immune related adverse events, including cardiotoxicity [5] that may be serious and have a poor prognosis, such as myocarditis, pericardial disease [5], non-inflammatory left ventricular dysfunction [6] and myocardial infarction (MI) [7]. Adverse cardiac events caused by ICIs occur at a low rate but can be accompanied by life-threatening events. Studies have shown that the mortality of affected patients remains as high as 50% [8, 9]. Although people are more and more aware of the cardiotoxicity caused by ICIs in the treatment of lung cancer, its incidence rate has not been systematically analyzed.

For cancer survivors, asymptomatic or symptomatic treatment related cardiac dysfunction or cardiac abnormalities may be responsible for interruption or discontinuation of cancer-directed therapies, which may reduce the chance for long-term survival [10]. By analyzing all published randomized clinical trials (RCTs) on ICIs, this study aims to evaluate the incidence of cardiotoxicity related to the ICI therapies for lung cancer, so as to enhance clinicians' attention to cardiotoxicity, implement proper prevention and intervention for high-risk patients, and minimize the risk of cardiac dysfunction during and after completion of therapy.

## Methods

The study was registered with INPLASY202250042 (https://inplasy.com/inplasy-2022-5-0042/) and reported in accordance with the PRISMA statement [11].

### Search strategy and selection criteria

We conducted a systematic literature search for relevant publications in PubMed and Scopus from inception to 19 April 2022. Review articles, case series, conference abstracts, and articles not published in English were excluded. The full search strategies are supplied in Additional file 1: M1. Additional articles were identified through reference lists and relevant systematic reviews. We considered all randomized studies on ICIs for lung cancer. Studies were eligible if they reported outcome data with regards to immune related adverse events. Observational studies were not considered.

### Study selection and data extraction

The study selection and data extraction were performed by two authors independently. Disagreements were resolved through discussion. Data were extracted, including first author, publication year, study design, study registration, treatments, sample size in each arm, tumor type and stage, follow-up time, outcome measures. The primary outcome of this meta-analysis was the risk ratio of any cardiotoxicity between two ICI-related therapies (including Single-ICI vs Chemotherapy, Single-ICI + Chemotherapy vs Chemotherapy, and Single-ICI vs Dual-ICl). The secondary outcomes were incidence of ICI-associated myocarditis, pericardial effusion, heart failure, cardiopulmonary events, cardiac arrest, atrial fibrillation, arrhythmia, and MI. Risks of bias were assessed independently using the Risk of Bias Tool developed by the Cochrane Collaboration [12].

### Statistical analysis

The incidence of cardiotoxicity may be very rare, even no event occurring in either or both arms of a study. Meta-analysis of incidence using inverse variance methods has the problem that the variance becomes very small when the incidence is small or large, with the consequence that such studies get a large weight in the meta-analysis. Transformation methods can be used to avoid an undue large weight for studies with small or large incidence. The double arcsine transformation [13] has properties that make it the clearly preferred option over the often-used logit transformation. Pooled incidence and risk ratios (RRs) with 95% confidence intervals (95% CIs) for cardiotoxicity events were calculated. This meta-analysis was conducted in MetaXL 5.3 (EpiGear International) using the IVhet (inverse variance heterogeneity) model [14]. The Chi^2^ test and the Higgins I^2^ statistics were used to assess heterogeneity between the included studies [15]. In addition, sensitivity analyses were performed by a leave-one-out analysis. Publication bias was assessed with the LFK index and Doi plot. The Interpretation of the index in terms of asymmetry was in Additional file 1: M2.

## Results

### Study characteristics

**Fig. 1:**
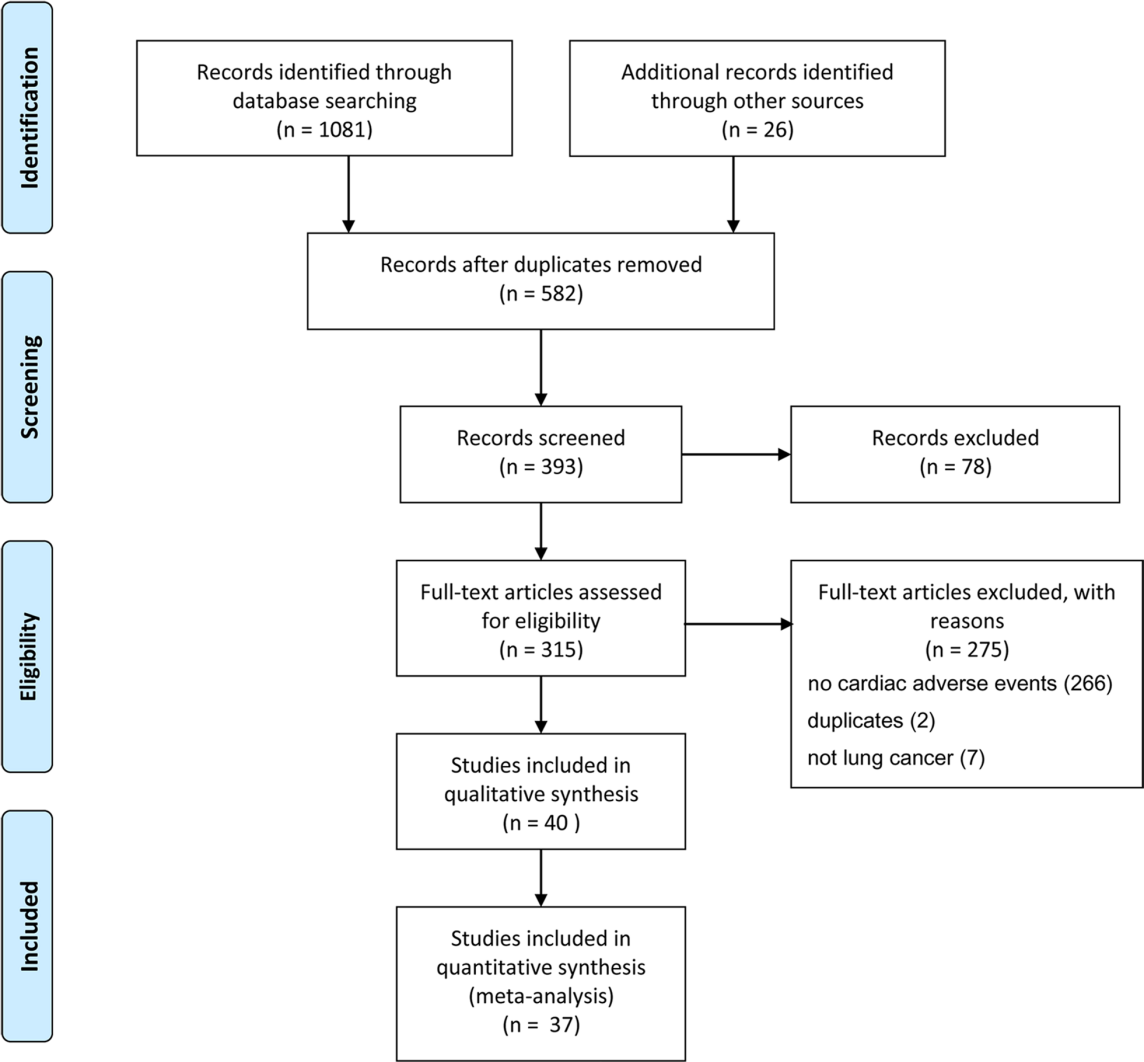
Flowchart of study selection

Our literature search returned 1081 articles, of which 315 were assessed as eligible. A total of 37 studies covering 38 trials, including 14,342 patients, were identified to be based on quantitative analyses (Fig. 1). Among them, six trials were phase 1a/b study, fourteen trials were phase 2 study, and eighteen trials were phase 3 study. Nine trials covered patients with small-cell lung cancer (SCLC) and 29 trials reported patients with nonsmall-cell lung cancer (NSCLC). Nineteen trials reported cardiac adverse events (AEs) with single ICI, twelve trials reported cardiac AEs with single ICI plus chemotherapy, and seven trials reported cardiac AEs with dual ICI plus or minus radiotherapy. Seven trials provided data on cardiac AEs of only ICI versus chemotherapy, nine trials provided data on cardiac AEs of single ICI versus single ICI plus chemotherapy, and four trials provided data on single ICI versus dual ICI. The characteristics of each study are shown in Table 1. Additional file 1: Fig. S1 and S2 describe the risk of bias according to each study and a summary of the risk of bias, respectively. Except for five trials, namely KEYNOTE-598 [16], KEYNOTE-189 [17], PACIFIC [18], IMpower133 [19], and CA184-156 [20] was a double- blind trial, the other 32 trials were open label trials. The risk of attrition bias exists in seven trials due to small sample size.

**Table 1:** Characteristics of included studies

First author	Year	Study	Study design	Phase	Tumor type	Treatments	Sample size	Median follow-up (month)
Altorki [21]	2021	NCT02904954	single-centre, open-label, randomised, controlled,	2	clinical stages I-IIIA NSCLC	neoadjuvant durvalumab alone versus neoadjuvant durvalumab + stereotactic radiotherapy	60	16.9
Antonia [22]	2016	NCT01928394	multicentre, open-label	1/2	limited-stage or extensive-stage SCLC, had disease progression after at least one previous platinum-containing regimen	Nivolumab versus Nivolumab + ipilimumab	213	198.5 days
Bang [23]	2020	JVDJ	single-arm, non-randomised, multi-cohort	Ia/b	advanced NSCLC	Ramucirumab	28	22.6
Boyer [16]	2021	KEYNOTE-598 NCT03302234	randomized, double-blind,	3	Metastatic NSCLC PDL1 tumor proportion score > = 50%	Pembrolizumab versus Pembrolizumab + ipilimumab	568	24.0
Felip [24]	2020	-	open-label, multicenter, dose-escalation and expansion	1b	stage IIIB/IV ALK-rearranged NSCLC	Ceritinib + Nivolumab	36	24.6
Garassino [25]	2018	NCT02087423	open-label, single-arm	2	advanced NSCLC	Durvalumab	444	12.0
Gettinger [26]	2021	Lung-MAP S1400I NCT02785952	open-label randomized	3	previously treated patients with Stage IV squamous Cell Lung Cancer	Nivolumab + ipilimumab versus Nivolumab	246	29.5
Herbst [27]	2021	JVDF NCT02443324	multicohort, non-randomized, open-label,	1a/b	treatment-naïve, locally advanced unresectable or metastatic NSCLC	ramucirumab + pembrolizumab	26	23.5
Hui [28]	2017	KEYNOTE-001 NCT01295827	international, randomized, open-labe	1	advanced NSCLC	Pembrolizumab	101	22.2
Ikeda [29]	2020	TORG1936 /AMBITIOUS jRCTs031190084	multicenter, single-arm	2	NSCLC with idiopathic interstitial pneumonias	atezolizumab	17	3.0
Jotte [30]	2020	IMpower131 NCT02367794	global, open-labe	3	stage IV squamous NSCLC	atezolizumab + carboplatin + paclitaxel versus atezolizumab + carboplatin + nabpaclitaxel versus carboplatin + nab-paclitaxel	1000	18.1
Juergens [31]	2020	NCT02537418	multicenter multi-cohort	1b	advanced, metastatic, recurrent or unresectable SCLC	durvalumab + tremelimumab + chemotherapy	22	19.6
Kanda [32]	2016	(JapicCTI)-132,071	single-center, open-label	1b	stage IIIB without indication for definitive radiotherapy, stage IV, or recurrent NSCLC	nivolumab + chemotherapy	24	6
Langer [33]	2016	KEYNOTE-021 NCT02039674	randomised, open-label	2	advanced NSCLC	Pembrolizumab + chemotherapy versus Chemotherapy	123	10·6
Lin [34]	2020	DETERRED NCT02525757	randomised, open-label	2	non-metastatic and unresectable NSCLC	atezolizumab	40	15.3
Malhotra [35]	2021	NCT03026166	multicenter, open-label	1–2	Previously Treated Extensive-Stage SCLC	Rova-T + nivolumab versus Rova-T + nivolumab and ilimumab	42	7.3
Mark [36]	2021	SAKK 19/17	multicenter, single-arm, and open-label trial	2	locally advanced, stage IIIB to IV, cytology or histology proven NSCLC	durvalumab	21	6.0
Mazieres [37]	2021	POPLAR NCT01903993	randomized, open-label	2	previously treated advanced NSCLC	Atezolizumab versus Docetaxel	277	48.0
Mazieres [37]	2021	OAK NCT02008227	randomized, open-label	3	previously treated advanced NSCLC	Atezolizumab versus Docetaxel	1187	48.0
Mok [38]	2019	KEYNOTE-042 NCT02220894	multicenter, randomized, open-label	3	previously untreated, PD-L1-expressing, locally advanced or metastatic NSCLC	Pembrolizumab versus Chemotherapy	1274	12·8
Nishio [39]	2021	IMpower132 NCT02657434	multicenter, randomized, openlabel	3	advanced NSCLC	atezolizumab + Chemotherapy versus Chemotherapy	101	17.5
Pakkala [40]	2020	NCT02701400	randomized, two-arm, non-comparative	2	relapsed SCLC	durvalumab(D) + tremelimumab(T) without SBRT versus SBRT followed D/T	18	5.7
Ramalingam [41]	2022	JASPER	multicenter, open-label	2	advanced (unresectable) or metastatic NSCLC (stage 3B/4)	Niraparib + Pembrolizumab	38	6.0
Rodríguez-Abreu [17]	2021	KEYNOTE-189 NCT02578680	double-blind trial	3	metastatic nonsquamous NSCLC without sensitizing EGFR/ ALK alterations	Pembrolizumab + chemotherapy versus Placebo + chemotherapy	607	31.0
Schoenfeld [42]	2022	NCT02888743	open-label, multicentre, randomised	2	metastatic NSCLC refractory to previous PD(L)-1 therapy	Durvalumab–tremelimumab ± radiotherapy	78	12·4
Sezer [43]	2021	EMPOWER-Lung 1 NCT03088540	multicentre, open-label, global	3	advanced NSCLC	Cemiplimab versus Chemotherapy	697	13·1
Welsh [44]	2020	NCT02444741	prospective randomized	1/2	metastatic NSCLC	Pembrolizumab with or without radiation therapy	100	20.4
Welsh [45]	2020	–	single-center, open-label	1/2	Limited-Stage SCLC	Pembrolizumab and chemoradiation	40	23.1
Antonia [18]	2017	PACIFIC	randomized, double-blind, international	3	stage III, locally advanced	Durvalumab versus placebo	709	14.5
Barlesi [46]	2018	JAVELIN Lung 200 NCT02395172	open-label, multicentre, randomised	3	stage IIIB, IV, or recurrent NSCLC with disease progression after previous platinum doublet treatment	Avelumab group versus Docetaxel	758	18.9
Borghaei [47]	2015	CheckMate-057 NCT01673867	randomized, open-label, international	3	stage IIIB or IV or recurrent nonsquamous NSCLC after radiation therapy or surgical resection	Nivolumab versus Docetaxel	555	–
Herbst [48]	2016	KEYNOTE-010	open-label, multicentre, randomised	2/3	previously treated, PD-L1-positive, advanced NSCLC	Pembrolizumab versus Pembrolizumab versus Docetaxel	991	13.1
Horn [19]	2018	IMpower133	double-blind, placebo-controlled,	3	Extensive-Stage SCLC	Atezolizumab versus Placebo	394	13.9
Paz-Ares [49]	2019	CASPIAN NCT03043872	open-label, multicentre, randomised	3	extensive-stage SCLC	Durvalumab + platinum– etoposide versus Platinum–etoposide	531	14.2
Reck [20]	2016	CA184-156 NCT01450761	multicenter, randomized, double-blind	3	Extensive-Stage SCLC	Chemotherapy/Ipilimumab versus Chemotherapy/Placebo	954	10.5
Socinski [50]	2018	Impower-150	international, randomised, open-label	3	Metastatic Nonsquamous NSCLC who had not previously received chemotherapy	atezolizumab + BCP versus bevacizumab + carboplatin + paclitaxel (BCP)	787	15.5
West [51]	2019	Impower-130	multicentre, randomised, open-label	3	metastatic NSCLC	Atezolizumab + chemotherapy versus Chemotherapy	705	19.2
Carbone [52]	2017	CheckMate-026	multicentre, randomised, open-label	3	Stage IV or Recurrent NSCLC	Nivolumab versus Chemotherapy	530	13.5
Total 38 studies							14,342	

*NSCLC* nonsmall-cell lung cancer, *SCLC* small-cell lung cancer

### Primary outcomes

#### Single ICI versus chemotherapy

The pooled RR of incidence of any cardiac AEs across the seven studies was 1.944 [95% CI 0.8–4.725], suggesting that the incidence of any cardiac AEs with single ICI treatment was 1.944 times higher than with chemotherapy, but was statistically insignificant (p-value = 0.142). I^2^ was 16%, indicating very small heterogeneity. Table 2, Fig. S3, Additional file 1: Table S1.

**Table 2:** Primary outcome: Comparison of incidence of cardiotoxicity between ICIS-related therapies and secondary outcomes: Incidence of various heart damages with ICIS-related therapies

Primary outcomes	RR	LCI 95%	HCI 95%	I-squared	P-value	Studies included	LFK index	Publication bias
Single-ICI vs chemotherapy	1.944	0.800	4.725	16.483	0.142	7	−1.6	*Minor*
Single-ICI + chemotherapy vs chemotherapy	***1.677***	***1.065***	***2.640***	***0.000***	***0.026***	***9***	***−1.77***	***Minor***
Single-ICI + CPA vs CPA	***1.877***	***1.121***	***3.143***			***3***		
Single-ICI + CPE vs CPE	2.666	0.546	13.011			3		
Single-ICI + CE vs CE	0.694	0.208	2.310			3		
Single-ICI vs dual-ICl	0.478	0.127	1.798	0.000	0.275	4	-1.81	*Minor*
Secondary outcomes	Incidence	LCI 95%	HCI 95%	I-squared	P-value	Studies included	LFK index	Publication bias
Single-ICI	0.007	0.001	0.015	80.550	–	19	3.96	Major
Single-ICI + chemotherapy	0.019	0.000	0.048	93.165	–	12	3.26	Major
Single-ICI + CPA	0.043	0.000	0.127			6		
Single-ICI + CPE	0.008	0.000	0.050			3		
Single-ICI + CE	0.004	0.000	0.011			3		
Dual-ICl	0.024	0.000	0.068	74.070	–	7	4.28	Major
Cardiotoxicity	0.012	0.004	0.023	87.985	–	38	4.22	Major
SCLC	0.010	0.000	0.024		–	9	–	–
NSCLC	0.013	0.003	0.026		–	29	–	–
Myocarditis	0.003	0.002	0.006	0.000	–	9	2.36	Major
Pericardial effusion	0.011	0.005	0.018	34.818	–	11	3.47	Major
Heart failure	0.006	0.003	0.010	29.494	–	10	4.65	Major
Cardiopulmonary events	0.003	0.001	0.007	9.267	–	5	1.64	***Minor***
Cardiac arrest	0.006	0.002	0.011	36.093	–	7	2.87	Major
Atrial fibrillation	0.009	0.000	0.031	87.712	–	9	3.74	Major
Arrhythmia	0.014	0.000	0.037	85.284	–	7	4.78	Major
Myocardial infarction	0.006	0.001	0.013	68.248	–	10	2.64	Major

Bold italic means significant difference

*CPA* cisplatin/carboplatin paclitaxel/nab paclitaxel, *CPE* cisplatin/carboplatin pemetrexed, *CE* cisplatin/carboplatin, etoposide, *RR* risk ratios. *LFK* index: A quantitative measure of Doi plot asymmetry called the LFK index (because it was developed by a graduate student, Luis Furuya-Kanamori), *No asymmetry* LFK index within ± 1, Minor asymmetry, LFK index exceeds ± 1 but within ± 2, Major asymmetry: LFK index exceeds ± 2

#### Single ICI plus chemotherapy versus chemotherapy

The pooled RR of incidence of any cardiac AEs across the nine studies was 1.677 [95%CI 1.065–2.64], suggesting that the incidence of any cardiac AEs with single ICI plus chemotherapy was 1.677 times higher than with chemotherapy, which was statistically significant (p-value = 0.026). I^2^ was 0%, indicating no heterogeneity. Table 2, Additional file 1: Fig. S4, Table S2.

#### Single ICI versus dual ICI

The pooled RR of incidence of any cardiac AEs across the four studies was 0.478 [95%CI 0.127–1.798], suggesting that the incidence of any cardiac AEs with single ICI was 47.8% of that with dual ICI, but was statistically insignificant (p-value = 0.275). I^2^ was 0%, indicating no heterogeneity. Table 2, Additional file 1: Fig. S5, Table S3.

### Secondary outcomes

The incidences of any cardiac AEs with single ICI, single ICI plus chemotherapy, and dual ICI plus or minus radiotherapy were 0.007 [95% CI 0.001–0.015], 0.019 [95% CI 0–0.048], and 0.024 [95% CI 0–0.068], respectively, showing that they were in an increasing trend. Table 2, Additional file 1: Fig. S6–S8. During ICI treatment, the incidence of myocarditis and arrhythmia was 0.003[95%CI 0.002–0.006] and 0.014[95%CI 0–0.037], respectively. The incidence of other cardiac damage was shown in Table 2, Additional file 1: Fig. S9–S16.

### Subgroup analyses

We divided the ICI related cardiac AEs into SCLC and NSCLC subgroups for meta-analysis. The incidence of ICI related cardiac AEs in SCLC subgroup was 0.010, while that in NSCLS subgroup was 0.013. Due to the lack of studies comparing ICI related cardiac AEs of these two types of lung cancer, it cannot be explained which subgroup has a significantly higher incidence. Table 2, Additional file 1: Fig. S17.

The subgroup analysis of Single-ICI + chemotherapy vs chemotherapy showed that the RR of cardiac toxicity of ICI + CPA vs CPA was 1.88 [1.12,3.14], indicating that the incidence of cardiotoxicity of ICI + CPA was 1.88 times higher than that of CPA, with statistical difference, while there was no statistical difference between ICI + CPE and CPE, or between ICI + CE and CE. Table 2. Additional file 1: Fig. S4.

### Sensitivity analyses

We performed a sensitivity analysis of all pooled results using leave-one-out analysis. When PACIFIC [18] was excluded, I^2^ decreased from 80 to 20%, and the incidence of any cardiac AEs with single ICI treatment decreased from 0.007 to 0.004, indicating that the heterogeneity of the pooled effect size (ES) mainly came from PACIFIC. When KEYNOTE-010 was excluded, I^2^ dropped from 80 to 0%, the pooled RR of incidence of any cardiac AEs with single ICI versus chemotherapy went from statistically insignificant 1.944[95%CI 0.8–4.725] to statistically significant 2.374 [95%CI 1.158–4.867]. This suggests that the heterogeneity of the pooled ES mainly came from KEYNOTE-010 [48], which altered the statistical significance of the pooled ES. When Impower-130 [51] was excluded, the pooled RR of incidence of any cardiac AEs with single ICI plus chemotherapy versus chemotherapy went from statistically significant 1.677 [95%CI 1.065–2.64] to statistically insignificant 1.257[95%CI 0.585–2.699], suggesting that the pooled ES were sensitive to Impower-130 [51], which altered the statistical significance of the pooled values. No sensitive studies were found in any of the other pooled ES.

### Publication bias

LFK index showed that there was major asymmetry and significant publication bias for the all results of "pooled incidence" except the pooled incidence of "cardiopulmonary". But the results of three comparisons (single ICI versus chemotherapy, single ICI plus chemotherapy versus chemotherapy, and single ICI versus dual ICI) showed minor asymmetry and publication bias. Additional file 1: Fig. S18–S32. Interpretation of the LFK index in terms of asymmetry see Additional file 1: M2.

## Discussion

A total of 38 studies involving 14,342 lung cancer patients were included in this meta-analysis. Our findings showed that there was no significant difference in the incidence of cardiotoxicity between single ICIs and chemotherapy alone, and that the increased risk of cardiotoxicity with combination immunotherapy versus single ICIs was statistically insignificant were fully consistent with the meta-analysis performed by Agostinetto et al. [53]. This should be good news for lung cancer patients. However, the incidence of cardiotoxicity with single ICIs and combination immunotherapy was 0.7% and 2.4%, respectively. This was also confirmed in an analysis of Vigibase (The World Health Organization’s international database of case safety reports) by Salem et al. [9], who observed that among 30,000 cancer patients treated with ICIs, combination immunotherapy exhibited a significantly higher rate of myocarditis (1.33%) than monotherapy did (0.31%). In addition, the mortality of myocarditis secondary to combination immunotherapy was higher than that of monotherapy (67% vs. 36%), suggesting that combination immunotherapy had a more severe myocarditis [54]. Similar findings were reported by Johnson et al. [55] in a query of the Bristol Myers Squibb Company safety database. We think this is due to the large differences of population and intervention between retrospective studies and RCTs. RCT's population is ideal for random assignment into groups that enjoy a similar baseline, whereas a retrospective study population comes from the real world and is susceptible to selection bias, and even if matching is performed, the results can be affected by various biases. “Pure” treatment in the RCT intervention and control groups are guaranteed in the best possible way to avoid exposure to other drugs, but in retrospective study all interventions are performed in clinical settings with a variety of comorbidities. Whatever, it's a real side of the real world. The results should therefore be interpreted with caution. Simultaneously, our study also found that ICIs plus chemotherapy increased the risk of cardiotoxicity by 67% compared with chemotherapy alone, suggesting that sometimes the combination is more cardiotoxic than monotherapy. Sensitivity analysis suggested that when removing IMpower-130 [51] changes the statistical significance. As can be seen from Figure S4, IMpower-130 is the study with the smallest confidence interval and significant weight in this comparison. So, this study had the largest impact on the pooled effect size. We believe that the weight assigned to IMpower-130 is reasonable using the IVhet model, and we prefer the pooled effect size with IMpower-130.

According to our pooled analysis of various cardiotoxicity, myocarditis showed the lowest incidence (0.3%), cardiac arrhythmia exhibited the highest incidence (1.4%), and the incidence of MI and pericardial effusion was 0.6% and 1.1%, respectively. Although the consequences of myocarditis and MI are serious, the high incidence of arrhythmia and pericardial disease cannot be ignored in clinical setting. Although the ICIs related cardiotoxicity mechanisms are currently unknown, there is a strong association between immune responses and heart disease. Severe systolic dysfunction/heart failure and fatal arrhythmias are often triggered by viral and autoimmune myocarditis. The heart is particularly vulnerable to immune related damage, and immune responses that normally lead to tissue damage and inflammation are particularly dangerous for the heart. The reason for this lies in its dense blood vessels that provide access to antibodies and immune cells, its anatomy is nonredundant and even small lesions can provide a substrate for arrhythmias [56]. Previous studies have demonstrated that PD-L1, PD-1, and CTLA-4 are important signaling pathways in cardiac immune crosstalk, and abrogation of those pathways leads to autoimmune myocarditis and heart failure [57, 58]. The independent autoantibody is the mechanism by which T cell-mediated responses to cardiac antigens promote disease progression and heart failure through myocardial inflammatory cell infiltration and increased myocardial fibrosis [59]. Collectively, acute MI, ventricular arrhythmias, autoimmune T cell-mediated myocarditis and conduction disease may be triggered by suppressing PD-L1, PD-1, or CTLA-4, and direct inhibition of PD-L1 may inevitably accelerate pre-existing heart disease, and invite noninflammatory cardiomyocyte dysfunction in diseased hearts even in the absence of an immune response.

In view of the severe cardiotoxicity with ICIs, detection of cardiac biomarkers in serum might be useful for baseline-based risk stratification, early diagnosis of cardiovascular disease during and after treatment, and identification of cancer patients who might benefit from cardioprotective therapy during continuing oncological treatment, as well as identifying patients with cardiovascular disease who might require long-term follow-up. Cardiac troponins (cTn) T and cTnI are structural proteins unique to the heart and are therefore organ-specific markers. Troponin assessment can help identify patients who may benefit from preventive treatment for cardiotoxicity and monitor response to cardioprotective therapy. We sought to perform a meta-analysis of serum biomarkers in patients receiving cardiotoxic cancer ICI therapies, by collecting as few as three relevant observational studies that provided some noteworthy results. According to. Mahmood et al. [8], among 35 ICI associated myocarditis patients and 105 ICI non-myocarditis patients, those who experienced major adverse cardiac events (MACE) obtained a higher admission, peak, and discharge/final troponin T value than those who did not. Patients with final/ discharge troponin T greater than or equal to 1.5 ng/ml were bound to a fourfold increased risk of MACE. Petricciuolo et al. [60] studied 30 patients who had high-sensitivity troponin T measured before starting ICI therapy. After 3 months of treatment, The MACE occurred only in 7 patients (23%) with high-sensitivity troponin T ≥ 14 ng/L at baseline. However, according to Yuan et al. [61], no significant changes in cTnI were found in a cohort of 19 cancer patients whose biomarkers were assessed at baseline, 1, 3, and 6 months after ICI administration. In our opinion, more studies are needed to determine whether cTn has the potential to be a serum biomarker for cardiotoxicity in ICI patients. Heart failure is a well-recognized complication that impacts survival and quality of life. It’s a progressive disorder [62]. This process begins with cardiotoxicity of immunotherapy and/or chemotherapy, and is usually progresses after structural change of the heart. It is increasingly important to address chronic and long-term adverse treatment effects in cancer survivors. For those high-risk lung cancer survivors, routine monitoring through cardiac imaging may be required after completion of lung cancer -directed therapy, in order to initiate appropriate interventions to prevent or even reverse the progression of cardiac dysfunction [10].

This meta-analysis has several limitations. For ethical reason in cancer treatment, the studies we included were basically from open label trials. In addition, there are also several options for chemotherapy or radiotherapy combined with ICI, and only one study was placebo-controlled. All these factors may lead to inter-study heterogeneity. Due to the extremely low incidence of cardiotoxicity, it is possible that no events will occur in any treatment arm, those studies with no cardiac events in all arms were excluded from pooling, the results of this meta-analysis might be overestimated. Due to the lack of adequate studies, there was no meta-analysis of serum biomarkers of cardiotoxicity in this study and no relevant conclusions were drawn.

## Conclusion

In summary, our study showed that single ICI did not increase the risk of cardiotoxicity compared with chemotherapy, and single ICI plus chemotherapy increased the risk of cardiotoxicity by 67% compared with chemotherapy alone. Our findings also suggested that combination immunotherapy did not increase the risk of cardiotoxicity compared with single ICI, and the conclusions of this meta-analysis should be interpreted with caution because of inconsistencies with the results of large retrospective studies.

## Supplementary Information

**Additional file 1: M1**. Search terms on PubMed. **M2** Interpretation of the index in terms of asymmetry. **Figure S1**. Risk of bias summary: review authors' judgements about each risk of bias item for each included study. **Figure S2**. Risk of bias graph: review authors' judgements about each risk of bias item presented as percentages across all included studies. **Figure S3**. Forest plot of rate ratio of any cardiac adverse events among patients with lung cancer, Single immune checkpoint inhibitor vs Chemotherapy. **Figure S4**. Forest plot of rate ratio of any cardiac adverse events among patients with lung cancer, Single immune checkpoint inhibitor +Chemotherapy vs Chemotherapy. **Figure S5**. Forest plot of rate ratio of any cardiac adverse events among patients with lung cancer, Single immune checkpoint inhibitor vs Dual immune checkpoint inhibitors. **Figure S6**. Forest plot of incidence of any cardiac adverse events in lung cancer patients treated with Single immune checkpoint inhibitor. **Figure S7**. Forest plot of incidence of any cardiac adverse events in lung cancer patients treated with Single immune checkpoint inhibitor plus Chemotherapy. **Figure S8**. Forest plot of incidence of any cardiac adverse events in lung cancer patients treated with Dual immune checkpoint inhibitors. **Figure S9**. Forest plot of incidence of myocarditis in lung cancer patients treated with immune checkpoint inhibitors. **Figure S10**. Forest plot of incidence of pericardial effusion in lung cancer patients treated with immune checkpoint inhibitors. **Figure S11**. Forest plot of incidence of heart failure in lung cancer patients treated with immune checkpoint inhibitors. **Figure S12**. Forest plot of incidence of cardiopulmonary events in lung cancer patients treated with immune checkpoint inhibitors. **Figure S13**. Forest plot of incidence of cardiac arrest in lung cancer patients treated with immune checkpoint inhibitors. **Figure S14**. Forest plot of incidence of atrial fibrillation in lung cancer patients treated with immune checkpoint inhibitors. **Figure S15**. Forest plot of incidence of arrhythmia in lung cancer patients treated with immune checkpoint inhibitors. **Figure S16**. Forest plot of incidence of Myocardial infarction in lung cancer patients treated with immune checkpoint inhibitors. **Figure S17**. Forest plot of incidence of any cardiac adverse events in lung cancer patients treated with immune checkpoint inhibitors. **Figure S18**. Doi plot of rate ratio of any cardiac adverse events among patients with lung cancer, Single immune checkpoint inhibitor vs Chemotherapy. **Figure S19**. Doi plot of rate ratio of any cardiac adverse events among patients with lung cancer, Single immune checkpoint inhibitor +Chemotherapy vs Chemotherapy. **Figure S20**. Doi plot of rate ratio of any cardiac adverse events among patients with lung cancer, Single immune checkpoint inhibitor vs Dual immune checkpoint inhibitors. **Figure S21**. Doi plot of incidence of any cardiac adverse events in lung cancer patients treated with Single immune checkpoint inhibitor. **Figure S22**. Doi plot of incidence of any cardiac adverse events in lung cancer patients treated with Single immune checkpoint inhibitor plus Chemotherapy. **Figure S23**. Doi plot of incidence of any cardiac adverse events in lung cancer patients treated with Dual immune checkpoint inhibitors. **Figure S24**. Doi plot of incidence of myocarditis in lung cancer patients treated with immune checkpoint inhibitors. **Figure S25**. Doi plot of incidence of pericardial effusion in lung cancer patients treated with immune checkpoint inhibitors. **Figure S26**. Doi plot of incidence of heart failure in lung cancer patients treated with immune checkpoint inhibitors. **Figure S27**. Doi plot of incidence of cardiopulmonary events in lung cancer patients treated with immune checkpoint inhibitors. **Figure S28**. Doi plot of incidence of cardiac arrest in lung cancer patients treated with immune checkpoint inhibitors. **Figure S29**. Doi plot of incidence of atrial fibrillation in lung cancer patients treated with immune checkpoint inhibitors. **Figure S30**. Doi plot of incidence of arrhythmia in lung cancer patients treated with immune checkpoint inhibitors. **Figure S31**. Doi plot of incidence of Myocardial infarction in lung cancer patients treated with immune checkpoint inhibitors. **Figure S32**. Doi plot of incidence of any cardiac adverse events in lung cancer patients treated with immune checkpoint inhibitors. **Table S1**. Detail of comparison of any cardiac adverse events among patients with lung cancer, Single immune checkpoint inhibitor vs Chemotherapy. **Table S2**. Detail of comparison of any cardiac adverse events among patients with lung cancer, Single immune checkpoint inhibitor +Chemotherapy vs Chemotherapy. **Table S3**. Detail of comparison of any cardiac adverse events among patients with lung cancer, Single immune checkpoint inhibitor vs Dual immune checkpoint inhibitors.

## Data Availability

All data generated or analysed during this study are included in this published article and its Additional information files.

## Abbreviations

ICIs: Immune checkpoint inhibitors
RCTs: Randomized clinical trials
MI: Myocardial infarction
RRs: Risk ratios
SCLC: Small-cell lung cancer
NSCLC: Nonsmall-cell lung cancer
AEs: Adverse events
ES: Effect size
cTn: Cardiac troponins
MACE: Major adverse cardiac events

## References

[1] Sung H, Ferlay J, Siegel RL (2021). Global cancer statistics 2020: GLOBOCAN estimates of incidence and mortality worldwide for 36 cancers in 185 Countries. CA Cancer J Clin.

[2] Doroshow DB, Sanmamed MF, Hastings K (2019). Immunotherapy in non-small cell lung cancer: facts and hopes. Clin Cancer Res.

[3] Ribas A, Wolchok JD (2018). Cancer immunotherapy using checkpoint blockade. Science.

[4] Haslam A, Prasad V (2019). Estimation of the percentage of US patients with cancer who are eligible for and respond to checkpoint inhibitor immunotherapy drugs. JAMA Netw Open.

[5] de Almeida DVP, Gomes JR, Haddad FJ, Buzaid AC (2018). Immune-mediated pericarditis with pericardial tamponade during nivolumab therapy. J Immunother.

[6] Roth ME, Muluneh B, Jensen BC, Madamanchi C, Lee CB (2016). Left ventricular dysfunction after treatment with ipilimumab for metastatic melanoma. Am J Ther.

[7] Weinstock C, Khozin S, Suzman D (2017). US food and drug administration approval summary: atezolizumab for metastatic non-small cell lung cancer. Clin Cancer Res..

[8] Mahmood SS, Fradley MG, Cohen JV (2018). Myocarditis in patients treated with immune checkpoint inhibitors. J Am Coll Cardiol.

[9] Salem JE, Manouchehri A, Moey M (2018). Cardiovascular toxicities associated with immune checkpoint inhibitors: an observational, retrospective, pharmacovigilance study. Lancet Oncol.

[10] Armenian SH, Lacchetti C, Barac A (2017). Prevention and monitoring of cardiac dysfunction in survivors of adult cancers: american society of clinical oncology clinical practice guideline. J Clin Oncol.

[11] Moher D, Liberati A, Tetzlaff J, Altman DG (2009). Preferred reporting items for systematic reviews and meta-analyses: the PRISMA statement. BMJ.

[12] Higgins JP, Altman DG, Gøtzsche PC (2011). The cochrane collaboration’s tool for assessing risk of bias in randomised trials. BMJ.

[13] Barendregt JJ, Doi SA, Lee YY, Norman RE, Vos T (2013). Meta-analysis of prevalence. J Epidemiol Commun Health.

[14] Doi SA, Barendregt JJ, Khan S, Thalib L, Williams GM (2015). Advances in the meta-analysis of heterogeneous clinical trials I: the inverse variance heterogeneity model. Contemp Clin Trials.

[15] Higgins JP, Thompson SG, Deeks JJ, Altman DG (2003). Measuring inconsistency in meta-analyses. BMJ.

[16] Boyer M, Şendur MAN, Rodríguez-Abreu D (2021). Pembrolizumab plus ipilimumab or placebo for metastatic non-small-cell lung cancer with PD-L1 tumor proportion score ≥ 50%: randomized, double-blind phase III KEYNOTE-598 study. J Clin Oncol.

[17] Rodríguez-Abreu D, Powell SF, Hochmair MJ (2021). Pemetrexed plus platinum with or without pembrolizumab in patients with previously untreated metastatic nonsquamous NSCLC: protocol-specified final analysis from KEYNOTE-189. Ann Oncol.

[18] Antonia SJ, Villegas A, Daniel D (2017). Durvalumab after chemoradiotherapy in stage iii non-small-cell lung cancer. N Engl J Med.

[19] Horn L, Mansfield AS, Szczęsna A (2018). First-line atezolizumab plus chemotherapy in extensive-stage small-cell lung cancer. N Engl J Med.

[20] Reck M, Luft A, Szczesna A (2016). Phase III randomized trial of ipilimumab plus etoposide and platinum versus placebo plus etoposide and platinum in extensive-stage small-cell lung cancer. J Clin Oncol.

[21] Altorki NK, McGraw TE, Borczuk AC (2021). Neoadjuvant durvalumab with or without stereotactic body radiotherapy in patients with early-stage non-small-cell lung cancer: a single-centre, randomised phase 2 trial. Lancet Oncol.

[22] Antonia SJ, López-Martin JA, Bendell J (2016). Nivolumab alone and nivolumab plus ipilimumab in recurrent small-cell lung cancer (CheckMate 032): a multicentre, open-label, phase 1/2 trial. Lancet Oncol.

[23] Bang YJ, Golan T, Dahan L (2020). Ramucirumab and durvalumab for previously treated, advanced non-small-cell lung cancer, gastric/gastro-oesophageal junction adenocarcinoma, or hepatocellular carcinoma: an open-label, phase Ia/b study (JVDJ). Eur J Cancer.

[24] Felip E, de Braud FG, Maur M (2020). Ceritinib plus nivolumab in patients with advanced alk-rearranged non-small cell lung cancer: results of an open-label, multicenter, phase 1B study. J Thorac Oncol.

[25] Garassino MC, Cho BC, Kim JH (2018). Durvalumab as third-line or later treatment for advanced non-small-cell lung cancer (ATLANTIC): an open-label, single-arm, phase 2 study. Lancet Oncol.

[26] Gettinger SN, Redman MW, Bazhenova L (2021). Nivolumab plus ipilimumab vs nivolumab for previously treated patients with stage iv squamous cell lung cancer: the lung-MAP S1400I phase 3 randomized clinical trial. JAMA Oncol.

[27] Herbst RS, Arkenau HT, Bendell J (2021). Phase 1 expansion cohort of ramucirumab plus pembrolizumab in advanced treatment-naive NSCLC. J Thorac Oncol.

[28] Hui R, Garon EB, Goldman JW (2017). Pembrolizumab as first-line therapy for patients with PD-L1-positive advanced non-small cell lung cancer: a phase 1 trial. Ann Oncol.

[29] Ikeda S, Kato T, Kenmotsu H (2020). A phase 2 Study of atezolizumab for pretreated NSCLC with idiopathic interstitial pneumonitis. J Thorac Oncol.

[30] Jotte R, Cappuzzo F, Vynnychenko I (2020). Atezolizumab in combination with carboplatin and nab-paclitaxel in advanced squamous NSCLC (IMpower131): results from a randomized phase III trial. J Thorac Oncol.

[31] Juergens RA, Hao D, Ellis PM (2020). A phase IB study of durvalumab with or without tremelimumab and platinum-doublet chemotherapy in advanced solid tumours: canadian cancer trials group study IND226. Lung Cancer.

[32] Kanda S, Goto K, Shiraishi H (2016). Safety and efficacy of nivolumab and standard chemotherapy drug combination in patients with advanced non-small-cell lung cancer: a four arms phase Ib study. Ann Oncol.

[33] Langer CJ, Gadgeel SM, Borghaei H (2016). Carboplatin and pemetrexed with or without pembrolizumab for advanced, non-squamous non-small-cell lung cancer: a randomised, phase 2 cohort of the open-label KEYNOTE-021 study. Lancet Oncol.

[34] Lin SH, Lin Y, Yao L (2020). Phase II trial of concurrent atezolizumab with chemoradiation for unresectable NSCLC. J Thorac Oncol.

[35] Malhotra J, Nikolinakos P, Leal T (2021). A phase 1–2 study of rovalpituzumab tesirine in combination with nivolumab plus or minus ipilimumab in patients with previously treated extensive-stage SCLC. J Thorac Oncol.

[36] Mark M, Froesch P, Eboulet EI (2021). SAKK 19/17: safety analysis of first-line durvalumab in patients with PD-L1 positive, advanced nonsmall cell lung cancer and a performance status of 2. Cancer Immunol Immunother.

[37] Mazieres J, Rittmeyer A, Gadgeel S (2021). Atezolizumab versus docetaxel in pretreated patients with NSCLC: final results from the randomized phase 2 POPLAR and phase 3 OAK clinical trials. J Thorac Oncol.

[38] Mok TSK, Wu YL, Kudaba I (2019). Pembrolizumab versus chemotherapy for previously untreated, PD-L1-expressing, locally advanced or metastatic non-small-cell lung cancer (KEYNOTE-042): a randomised, open-label, controlled, phase 3 trial. Lancet.

[39] Nishio M, Saito H, Goto K (2021). IMpower132: Atezolizumab plus platinum-based chemotherapy vs chemotherapy for advanced NSCLC in Japanese patients. Cancer Sci.

[40] Pakkala S, Higgins K, Chen Z (2020). Durvalumab and tremelimumab with or without stereotactic body radiation therapy in relapsed small cell lung cancer: a randomized phase II study. J Immunother Cancer.

[41] Ramalingam SS, Thara E, Awad MM (2022). JASPER: Phase 2 trial of first-line niraparib plus pembrolizumab in patients with advanced non-small cell lung cancer. Cancer.

[42] Schoenfeld JD, Giobbie-Hurder A, Ranasinghe S (2022). Durvalumab plus tremelimumab alone or in combination with low-dose or hypofractionated radiotherapy in metastatic non-small-cell lung cancer refractory to previous PD(L)-1 therapy: an open-label, multicentre, randomised, phase 2 trial. Lancet Oncol.

[43] Sezer A, Kilickap S, Gümüş M (2021). Cemiplimab monotherapy for first-line treatment of advanced non-small-cell lung cancer with PD-L1 of at least 50%: a multicentre, open-label, global, phase 3, randomised, controlled trial. Lancet.

[44] Welsh J, Menon H, Chen D (2020). Pembrolizumab with or without radiation therapy for metastatic non-small cell lung cancer: a randomized phase I/II trial. J Immunother Cancer.

[45] Welsh JW, Heymach JV, Guo C (2020). Phase 1/2 trial of pembrolizumab and concurrent chemoradiation therapy for limited-stage SCLC. J Thorac Oncol.

[46] Barlesi F, Vansteenkiste J, Spigel D (2018). Avelumab versus docetaxel in patients with platinum-treated advanced non-small-cell lung cancer (JAVELIN Lung 200): an open-label, randomised, phase 3 study. Lancet Oncol.

[47] Borghaei H, Paz-Ares L, Horn L (2015). Nivolumab versus docetaxel in advanced nonsquamous non-small-cell lung cancer. N Engl J Med.

[48] Herbst RS, Baas P, Kim DW (2016). Pembrolizumab versus docetaxel for previously treated, PD-L1-positive, advanced non-small-cell lung cancer (KEYNOTE-010): a randomised controlled trial. Lancet.

[49] Paz-Ares L, Dvorkin M, Chen Y (2019). Durvalumab plus platinum-etoposide versus platinum-etoposide in first-line treatment of extensive-stage small-cell lung cancer (CASPIAN): a randomised, controlled, open-label, phase 3 trial. Lancet.

[50] Socinski MA, Jotte RM, Cappuzzo F (2018). Atezolizumab for first-line treatment of metastatic nonsquamous NSCLC. N Engl J Med.

[51] West H, McCleod M, Hussein M (2019). Atezolizumab in combination with carboplatin plus nab-paclitaxel chemotherapy compared with chemotherapy alone as first-line treatment for metastatic non-squamous non-small-cell lung cancer (IMpower130): a multicentre, randomised, open-label, phase 3 trial. Lancet Oncol.

[52] Carbone DP, Reck M, Paz-Ares L (2017). First-line nivolumab in stage iv or recurrent non-small-cell lung cancer. N Engl J Med.

[53] Agostinetto E, Eiger D, Lambertini M (2021). Cardiotoxicity of immune checkpoint inhibitors: A systematic review and meta-analysis of randomised clinical trials. Eur J Cancer.

[54] Moslehi JJ, Salem JE, Sosman JA, Lebrun-Vignes B, Johnson DB (2018). Increased reporting of fatal immune checkpoint inhibitor-associated myocarditis. Lancet.

[55] Johnson DB, Balko JM, Compton ML (2016). Fulminant Myocarditis with Combination Immune Checkpoint Blockade. N Engl J Med.

[56] Moslehi J, Lichtman AH, Sharpe AH, Galluzzi L, Kitsis RN (2021). Immune checkpoint inhibitor-associated myocarditis: manifestations and mechanisms. J Clin Invest.

[57] Lichtman AH (2013). The heart of the matter: protection of the myocardium from T cells. J Autoimmun.

[58] Tarrio ML, Grabie N, Bu DX, Sharpe AH, Lichtman AH (2012). PD-1 protects against inflammation and myocyte damage in T cell-mediated myocarditis. J Immunol.

[59] Gröschel C, Sasse A, Röhrborn C (2017). T helper cells with specificity for an antigen in cardiomyocytes promote pressure overload-induced progression from hypertrophy to heart failure. Sci Rep.

[60] Petricciuolo S, Delle Donne MG, Aimo A, Chella A, De Caterina R (2021). Pre-treatment high-sensitivity troponin T for the short-term prediction of cardiac outcomes in patients on immune checkpoint inhibitors. Eur J Clin Invest.

[61] Yuan M, Zang L, Xu A (2021). Dynamic changes of serum heart type-fatty acid binding protein in cancer patients treated with immune checkpoint inhibitors. Front Pharmacol.

[62] Yancy CW, Jessup M, Bozkurt B (2013). 2013 ACCF/AHA guideline for the management of heart failure: a report of the american college of cardiology foundation/american heart association task force on practice guidelines. Circulation.

