# Supplementary material for: Immune checkpoint inhibitor-related adverse cardiac events in patients with lung cancer: a systematic review and meta-analysis
Source: Cancer Cell Int. 2022 Nov 19;22:363. doi: 10.1186/s12935-022-02760-2 (PMC9675058; doi:10.1186/s12935-022-02760-2)
Supplement: Supplementary file 1 — Additional file 1: M1. Search terms on PubMed. M2 Interpretation of the index in terms of asymmetry. Figure S1. Risk of bias summary: review authors' judgements about each risk of bias item for each included study. Figure S2. Risk of bias graph: review authors' judgements about each risk of bias item presented as percentages across all included studies. Figure S3. Forest plot of rate ratio of any cardiac adverse events among patients with lung cancer, Single immune checkpoint inhibitor vs Chemotherapy. Figure S4. Forest plot of rate ratio of any cardiac adverse events among patients with lung cancer, Single immune checkpoint inhibitor +Chemotherapy vs Chemotherapy. Figure S5. Forest plot of rate ratio of any cardiac adverse events among patients with lung cancer, Single immune checkpoint inhibitor vs Dual immune checkpoint inhibitors. Figure S6. Forest plot of incidence of any cardiac adverse events in lung cancer patients treated with Single immune checkpoint inhibitor. Figure S7. Forest plot of incidence of any cardiac adverse events in lung cancer patients treated with Single immune checkpoint inhibitor plus Chemotherapy. Figure S8. Forest plot of incidence of any cardiac adverse events in lung cancer patients treated with Dual immune checkpoint inhibitors. Figure S9. Forest plot of incidence of myocarditis in lung cancer patients treated with immune checkpoint inhibitors. Figure S10. Forest plot of incidence of pericardial effusion in lung cancer patients treated with immune checkpoint inhibitors. Figure S11. Forest plot of incidence of heart failure in lung cancer patients treated with immune checkpoint inhibitors. Figure S12. Forest plot of incidence of cardiopulmonary events in lung cancer patients treated with immune checkpoint inhibitors. Figure S13. Forest plot of incidence of cardiac arrest in lung cancer patients treated with immune checkpoint inhibitors. Figure S14. Forest plot of incidence of atrial fibrillation in lung cancer patients treated with imm [file 12935_2022_2760_MOESM1_ESM.docx]

**Additional Information**

**M1** Search terms on PubMed

**M2** Interpretation of the index in terms of asymmetry:

**Figure S1** Risk of bias summary: review authors' judgements about each risk of bias item for each included study.

**Figure S2** Risk of bias graph: review authors' judgements about each risk of bias item presented as percentages across all included studies.

**Figure S3** Forest plot of rate ratio of any cardiac adverse events among patients with lung cancer, Single immune checkpoint inhibitor vs Chemotherapy

**Table S1** Detail of comparison of any cardiac adverse events among patients with lung cancer, Single immune checkpoint inhibitor vs Chemotherapy

**Figure S4** Forest plot of rate ratio of any cardiac adverse events among patients with lung cancer, Single immune checkpoint inhibitor +Chemotherapy vs Chemotherapy

**Table S2** Detail of comparison of any cardiac adverse events among patients with lung cancer, Single immune checkpoint inhibitor +Chemotherapy vs Chemotherapy

**Figure S5** Forest plot of rate ratio of any cardiac adverse events among patients with lung cancer, Single immune checkpoint inhibitor vs Dual immune checkpoint inhibitors

**Table S3** Detail of comparison of any cardiac adverse events among patients with lung cancer, Single immune checkpoint inhibitor vs Dual immune checkpoint inhibitors

**Figure S6** Forest plot of incidence of any cardiac adverse events in lung cancer patients treated with Single immune checkpoint inhibitor

**Figure S7** Forest plot of incidence of any cardiac adverse events in lung cancer patients treated with Single immune checkpoint inhibitor plus Chemotherapy

**Figure S8** Forest plot of incidence of any cardiac adverse events in lung cancer patients treated with Dual immune checkpoint inhibitors

**Figure S9** Forest plot of incidence of myocarditis in lung cancer patients treated with immune checkpoint inhibitors

**Figure S10** Forest plot of incidence of pericardial effusion in lung cancer patients treated with immune checkpoint inhibitors

**Figure S11** Forest plot of incidence of heart failure in lung cancer patients treated with immune checkpoint inhibitors

**Figure S12** Forest plot of incidence of cardiopulmonary events in lung cancer patients treated with immune checkpoint inhibitors

**Figure S13** Forest plot of incidence of cardiac arrest in lung cancer patients treated with immune checkpoint inhibitors

**Figure S14** Forest plot of incidence of atrial fibrillation in lung cancer patients treated with immune checkpoint inhibitors

**Figure S15** Forest plot of incidence of arrhythmia in lung cancer patients treated with immune checkpoint inhibitors

**Figure S16** Forest plot of incidence of Myocardial infarction in lung cancer patients treated with immune checkpoint inhibitors

**Figure S17** Forest plot of incidence of any cardiac adverse events in lung cancer patients treated with immune checkpoint inhibitors

**Figure S18** Doi plot of rate ratio of any cardiac adverse events among patients with lung cancer, Single immune checkpoint inhibitor vs Chemotherapy

**Figure S19** Doi plot of rate ratio of any cardiac adverse events among patients with lung cancer, Single immune checkpoint inhibitor +Chemotherapy vs Chemotherapy

**Figure S20** Doi plot of rate ratio of any cardiac adverse events among patients with lung cancer, Single immune checkpoint inhibitor vs Dual immune checkpoint inhibitors

**Figure S21** Doi plot of incidence of any cardiac adverse events in lung cancer patients treated with Single immune checkpoint inhibitor

**Figure S22** Doi plot of incidence of any cardiac adverse events in lung cancer patients treated with Single immune checkpoint inhibitor plus Chemotherapy

**Figure S23** Doi plot of incidence of any cardiac adverse events in lung cancer patients treated with Dual immune checkpoint inhibitors

**Figure S24** Doi plot of incidence of myocarditis in lung cancer patients treated with immune checkpoint inhibitors

**Figure S25** Doi plot of incidence of pericardial effusion in lung cancer patients treated with immune checkpoint inhibitors

**Figure S26** Doi plot of incidence of heart failure in lung cancer patients treated with immune checkpoint inhibitors

**Figure S27** Doi plot of incidence of cardiopulmonary events in lung cancer patients treated with immune checkpoint inhibitors

**Figure S28** Doi plot of incidence of cardiac arrest in lung cancer patients treated with immune checkpoint inhibitors

**Figure S29** Doi plot of incidence of atrial fibrillation in lung cancer patients treated with immune checkpoint inhibitors

**Figure S30** Doi plot of incidence of arrhythmia in lung cancer patients treated with immune checkpoint inhibitors

**Figure S31** Doi plot of incidence of Myocardial infarction in lung cancer patients treated with immune checkpoint inhibitors

**Figure S32** Doi plot of incidence of any cardiac adverse events in lung cancer patients treated with immune checkpoint inhibitors

**M1** Search terms on PubMed

(('lung cancer'[Title/Abstract]) OR ('NSCLC'[Title/Abstract]) OR ('SCLC'[Title/Abstract])) AND (('Atezolizumab' [Title/Abstract]) OR ('Avelumab' [Title/Abstract]) OR ('Nivolumab'[Title/Abstract]) OR ('BMS936559' [Title/Abstract]) OR ('BMS-936559' [Title/Abstract]) OR ('Durvalumab' [Title/Abstract]) OR ('Ipilimumab' [Title/Abstract]) OR ('Pembrolizumab'[Title/Abstract])OR ('Pidilizumab' [Title/Abstract]) OR ('Tremelimumab' [Title/Abstract]) OR ('Spartalizumab' [Title/Abstract]) OR ('Cemiplimab' [Title/Abstract]))

**M2** Interpretation of the index in terms of asymmetry

A quantitative measure of Doi plot asymmetry called the LFK index (because it was developed by a graduate student, Luis Furuya-Kanamori)

No asymmetry: LFK index within ±1

Minor asymmetry: LFK index exceeds ±1 but within ±2

Major asymmetry: LFK index exceeds ±2

**Figure S1**. Risk of bias summary: review authors' judgements about each risk of bias item for each included study.


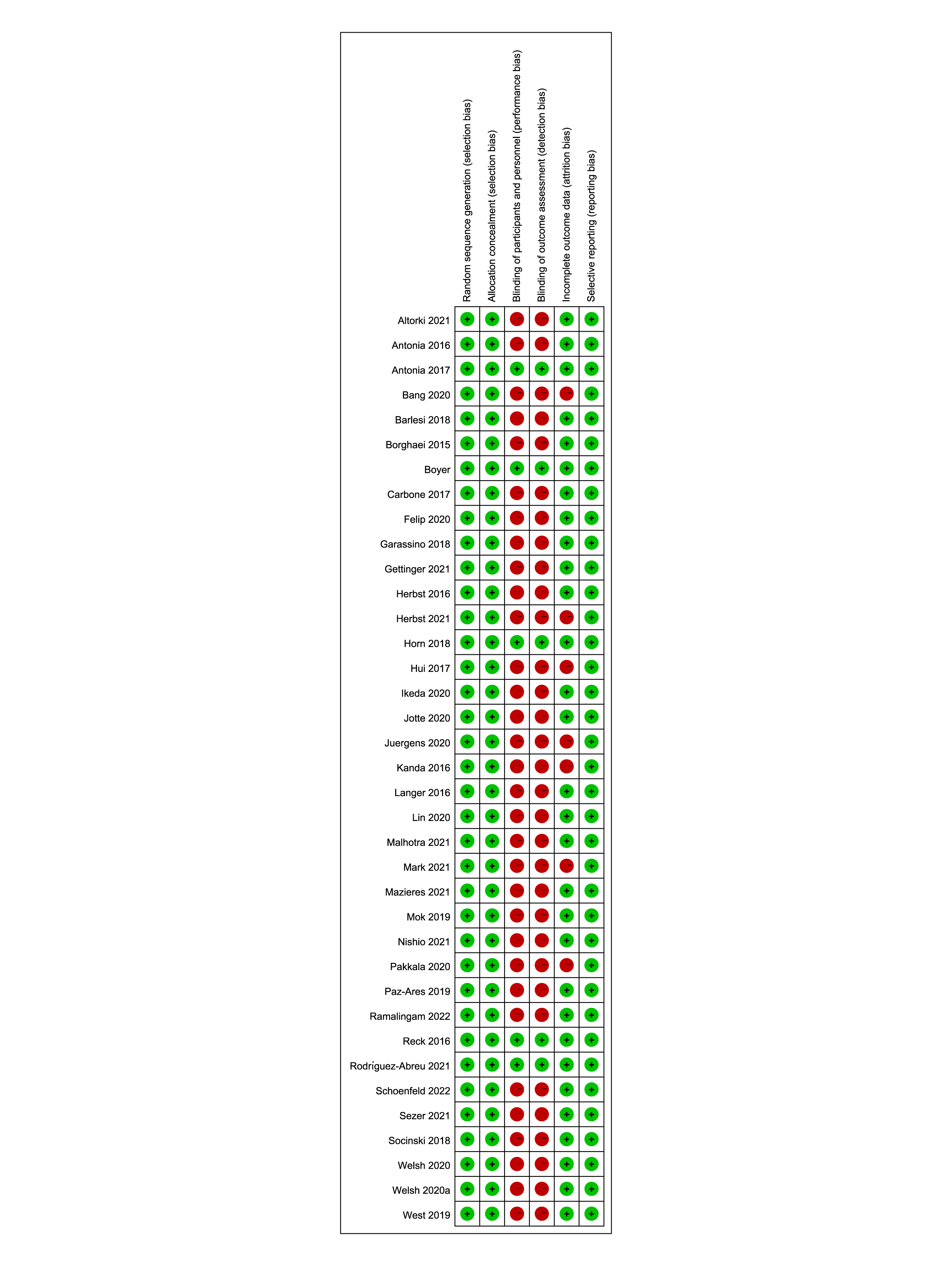


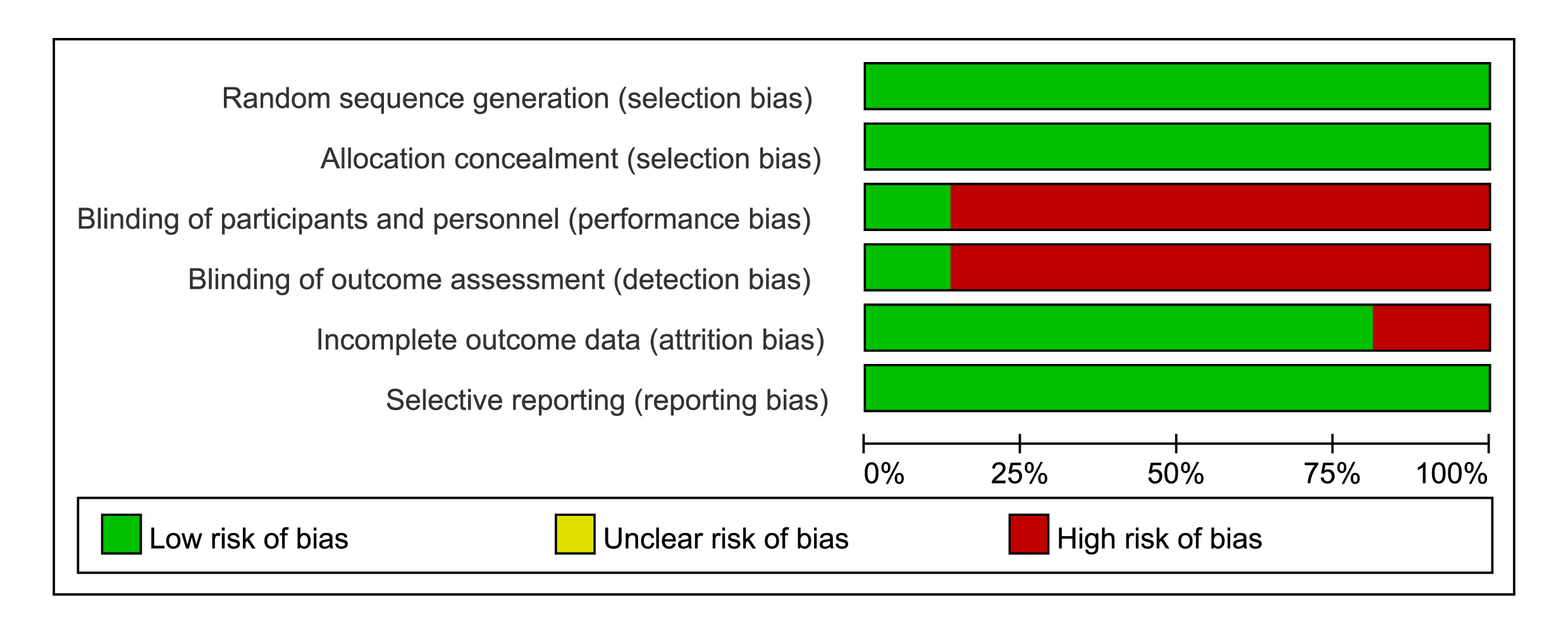
**Figure S2.** Risk of bias graph: review authors' judgements about each risk of bias item presented as percentages across all included studies.

**Figure S3**. Forest plot of rate ratio of any cardiac adverse events among patients with lung cancer, Single immune checkpoint inhibitor vs Chemotherapy

**Table S1.** Detail of comparison of any cardiac adverse events among patients with lung cancer, Single immune checkpoint inhibitor vs Chemotherapy

| Study | One-ICI | | Chemotherapy | |
| --- | --- | --- | --- | --- |
|  | N | Cases | N | Cases |
| KEYNOTE-042 | 636 | 1 | 615 | 0 |
| EMPOWER-Lung 1 | 355 | 5 | 342 | 1 |
| PACIFIC | 475 | 28 | 234 | 5 |
| JAVELIN Lung 200 | 393 | 2 | 365 | 2 |
| CheckMate-057 | 287 | 1 | 268 | 0 |
| KEYNOTE-010 | 682 | 0 | 309 | 3 |
| CheckMate-026 | 267 | 1 | 263 | 1 |

**Figure S4**. Forest plot of rate ratio of any cardiac adverse events among patients with lung cancer, Single immune checkpoint inhibitor +Chemotherapy vs Chemotherapy

**Table S2.** Detail of comparison of any cardiac adverse events among patients with lung cancer, Single immune checkpoint inhibitor +Chemotherapy vs Chemotherapy

| Study | One-ICI + Chemotherapy | | Chemotherapy | |
| --- | --- | --- | --- | --- |
|  | N | Cases | N | Cases |
| IMpower131 | 332 | 1 | 334 | 1 |
| KEYNOTE-021 | 59 | 1 | 62 | 0 |
| IMpower132 | 48 | 3 | 52 | 1 |
| KEYNOTE-189 | 405 | 1 | 202 | 0 |
| IMpower133 | 198 | 0 | 196 | 1 |
| CASPIAN | 265 | 3 | 266 | 5 |
| CA184-156 | 478 | 1 | 476 | 0 |
| Impower-150 | 393 | 5 | 394 | 3 |
| Impower-130 | 473 | 56 | 232 | 14 |

**Figure S5**. Forest plot of rate ratio of any cardiac adverse events among patients with lung cancer, Single immune checkpoint inhibitor vs Dual immune checkpoint inhibitors

**Table S3.** Detail of comparison of any cardiac adverse events among patients with lung cancer, Single immune checkpoint inhibitor vs Dual immune checkpoint inhibitors

| Study name | One-ICI | | Dual-ICI | |
| --- | --- | --- | --- | --- |
|  | N | Cases | N | Cases |
| NCT01928394 | 98 | 1 | 61 | 1 |
| KEYNOTE-598 | 281 | 0 | 282 | 2 |
| Lung-MAP S1400I | 123 | 0 | 124 | 3 |
| NCT03026166 | 30 | 3 | 12 | 1 |

**Figure S6**. Forest plot of incidence of any cardiac adverse events in lung cancer patients treated with Single immune checkpoint inhibitor

**Figure S7**. Forest plot of incidence of any cardiac adverse events in lung cancer patients treated with Single immune checkpoint inhibitor plus Chemotherapy

**Figure S8**. Forest plot of incidence of any cardiac adverse events in lung cancer patients treated with Dual immune checkpoint inhibitors

**Figure S9**. Forest plot of incidence of myocarditis in lung cancer patients treated with immune checkpoint inhibitors

**Figure S10**. Forest plot of incidence of pericardial effusion in lung cancer patients treated with immune checkpoint inhibitors

**Figure S11**. Forest plot of incidence of heart failure in lung cancer patients treated with immune checkpoint inhibitors

**Figure S12**. Forest plot of incidence of cardiopulmonary events in lung cancer patients treated with immune checkpoint inhibitors

**Figure S13**. Forest plot of incidence of cardiac arrest in lung cancer patients treated with immune checkpoint inhibitors

**Figure S14**. Forest plot of incidence of atrial fibrillation in lung cancer patients treated with immune checkpoint inhibitors

**Figure S15**. Forest plot of incidence of arrhythmia in lung cancer patients treated with immune checkpoint inhibitors

**Figure S16**. Forest plot of incidence of Myocardial infarction in lung cancer patients treated with immune checkpoint inhibitors

**Figure S17**. Forest plot of incidence of any cardiac adverse events in lung cancer patients treated with immune checkpoint inhibitors

**Figure S18**. Doi plot of rate ratio of any cardiac adverse events among patients with lung cancer, Single immune checkpoint inhibitor vs Chemotherapy

**Figure S19**. Doi plot of rate ratio of any cardiac adverse events among patients with lung cancer, Single immune checkpoint inhibitor +Chemotherapy vs Chemotherapy

**Figure S20**. Doi plot of rate ratio of any cardiac adverse events among patients with lung cancer, Single immune checkpoint inhibitor vs Dual immune checkpoint inhibitors

**Figure S21**. Doi plot of incidence of any cardiac adverse events in lung cancer patients treated with Single immune checkpoint inhibitor

**Figure S22**. Doi plot of incidence of any cardiac adverse events in lung cancer patients treated with Single immune checkpoint inhibitor plus Chemotherapy

**Figure S23**. Doi plot of incidence of any cardiac adverse events in lung cancer patients treated with Dual immune checkpoint inhibitors

**Figure S24**. Doi plot of incidence of myocarditis in lung cancer patients treated with immune checkpoint inhibitors

**Figure S25**. Doi plot of incidence of pericardial effusion in lung cancer patients treated with immune checkpoint inhibitors

**Figure S26** Doi plot of incidence of heart failure in lung cancer patients treated with immune checkpoint inhibitors

**Figure S27**. Doi plot of incidence of cardiopulmonary events in lung cancer patients treated with immune checkpoint inhibitors

**Figure S28**. Doi plot of incidence of cardiac arrest in lung cancer patients treated with immune checkpoint inhibitors

**Figure S29**. Doi plot of incidence of atrial fibrillation in lung cancer patients treated with immune checkpoint inhibitors

**Figure S30**. Doi plot of incidence of arrhythmia in lung cancer patients treated with immune checkpoint inhibitors

**Figure S31**. Doi plot of incidence of Myocardial infarction in lung cancer patients treated with immune checkpoint inhibitors

**Figure S32**. Doi plot of incidence of any cardiac adverse events in lung cancer patients treated with immune checkpoint inhibitors
